# Letter to the Baltimore Editor, from J. C. McCabe, of Richmond, Va.

**Published:** 1841

**Authors:** 


*Letter to the Baltimore Editor, from John C. McCabe, of Richmond, Va.*

DEAR DOCTOR,

The formation of a Society, similar in its objects to the American College of Dental Surgeons, has long been the theme of reflection, and its absence a source of regret with most of the best professors of the science of Dental Surgery; for, it has well been reasoned, until there is some public test of ability submitted by its professors, and evidence of their competency to discharge faithfully the duties of the art, imposition and fraud will have no bound to their progress; and impudent empiricism, and modest worth, will have the same seat appointed to them by the mass who cannot, in the very nature of things, make the distinction. If most of our Medical Colleges had among the various professorships, the Chair of Dental Surgery, and, as lecturers on that branch of science, men of known abilities, long since would dental surgery have taken that high stand which its importance so justly entitles it to, instead of being classed, as it is by some, as one of the humblest branches of surgery.

That Dental Surgery held a distinguished station among the important sciences with the ancients, we have only to turn to their various modes of treating the teeth, known to every educated dentist, for proof; and hence, if no other argument were necessary to enforce the establishment of a National College of Dentists, methinks the desire to rescue a noble science,—a science not unworthy the profound attention and study of the gifted ones of antiquity—from obloquy, would of itself form a powerful reason.

Ignorant pretenders, itinerating charlatans, impudent empirics, would be brought to a stand, and those who would dare question the claims of the worthy and intellectual, be silenced.

I cannot agree with the talented Dr. Parmly, that the mere name of Dental Surgeon ever did exclude from the refined circles of elegant hospitality its owner, or that it ever operated upon the professor of the science so as to exclude him from the festive board, or the refinements of the drawing room. There are men who will disgrace any profession

—and from such alone should the partialities of friendship and fellowship be kept. But there is a pre-eminence to which intellect entitles its possessor—a dignity which attaches itself to mind, which will always secure to the individual of any profession respect and deference; and whether it exhibit itself in the immortal songs of an Ayrshire ploughboy, as in the case of Burns, or gaze with the philosophic eye of a Franklin—himself a printer—or penetrate the canopy of the stars with the instrument maker, Rittenhosue, will ever command and compel admiration,—and the weak and squeamish fool who would object to a man purely on account of his profession, should be held up as a fair mark for the polished arrows of the satirist.

That our profession has been wofully disgraced, I make no question; I know the fact; but so has almost every other, from the sacred desk down to the nameless art. Let the professors of our science use their endeavours to elevate it, and it must, and will succeed. Many of them can wield a ready pen; let them contribute their mite towards the great treasury of information. New subjects present themselves every day in the course of our profession—let them be made known, let the curious and the scientific examine them,—let the different modes of our practice be criticised and compared: in short, let us strive to give to the profession that dignity and that character which it has had in past centuries, and which its importance so justly entitles it to, and to this end unite in the permanent establishment of "the American College of Dental Surgeons."

